# Downregulation of Interferon-β and Inhibition of TLR3 Expression are associated with Fatal Outcome of Severe Fever with Thrombocytopenia Syndrome

**DOI:** 10.1038/s41598-017-06921-6

**Published:** 2017-07-26

**Authors:** Peixin Song, Nan Zheng, Li Zhang, Yong Liu, Taoyu Chen, Changjun Bao, Zhifeng Li, Wei Yong, Yongyang Zhang, Chao Wu, Zhiwei Wu

**Affiliations:** 1 Department of Infectious Diseases, Nanjing Drum Tower Hospital, Nanjing University Medical School, Nanjing, PR China; 2 State Key Lab of Analytical Chemistry for Life Science, Nanjing University, Nanjing, PR China; 3 Center for Public Health Research, Nanjing University Medical School, Nanjing, PR China; 4 Department of Experimental Medicine, Nanjing Drum Tower Hospital, Nanjing University Medical School, Nanjing, PR China; 5 Jiangsu Provincial Center for Disease Control and Prevention, Nanjing, PR China; 6 Nanjing Center for Disease Control and Prevention, Nanjing, PR China

## Abstract

Severe Fever with Thrombocytopenia Syndrome (SFTS) is an emerging infectious disease with high mortality and increasing prevalence in the East Asia. Though the etiological agent has been identified as a novel Bunyavirus, cellular mechanisms of viral pathogenesis and host immune response to SFTS virus infection remain unknown. A comprehensive study was conducted on a cohort of 70 patients on clinical manifestations, viral loads, modulation of cytokines, serum interferon level, immune related gene expression in peripheral blood cells, and dynamic changes of circulating dendritic cells during the acute phase of SFTSV infection. We found that high level viremia, reduced platelets, coagulation dysfunction, multi-organ injuries, elevated IL-6 and TNF-α were closely associated with the aggravation of SFTS. In addition, we demonstrated strong correlations between disease severity and the decline of serum IFN-β and IL-1β level, reduction of myeloid dendritic cells (mDCs) and suppressed Toll like receptor 3 expression in monocytes and mDCs. In general, dysfunction of innate immune response and cytokine storm are both involved in the pathogenesis of SFTS. Reduction of myeloid DCs contributes to the fatal outcome of SFTS virus infection, and the regulation of TLR3 could probably be the mechanism.

## Introduction

Severe fever with thrombocytopenia syndrome (SFTS) is an emerging infectious disease characterized with high fever, thrombocytopenia, leukocytopenia, gastrointestinal symptoms, hemorrhage, and multiple organ failure, with high fatalities ranging from 12% to 30%^1–3^. There have been a number of epidemics in China, Japan and Korea^4, 5^ and the etiological agent has been identified as a novel member of Bunyaviridae^1^.

Recent research on the pathogenesis of SFTS virus infection mainly focused on two aspects: cytokine storm-mediated immune activation and mechanisms of impairment of innate immune response. Sun *et al*. reported that SFTS virus-induced cytokine storm was characterized by a drastic increase of IL-1RA, IL-6, IL-10, G-CSF, IP-10 and MCP-1 in the acute phase of infection, indicating that the immune activation contributes to the pathogenesis of SFTS^6^. Another study by Deng *et al*. showed similar results that serum TNF-α, IL-6, RANTES and IP-10 levels increased in SFTS patients, whereas serum level of IFN-γ decreased^7^. However, a study by Li *et al*. reported that IFN-γ levels were elevated in fatal cases, whereas TNF-α level decreased in both fatal and recovered patients^3^.

Cellular mechanisms involved in the suppression of type I interferon response had been investigated in THP-1 and other cell models^8, 9^. Jin *et al*. initially reported that in a mouse model, SFTS virus-bound platelets could be phagocytosed by monocyte-derived macrophages, in which effective replication of the virus was observed^10^. Further *in vitro* studies revealed that SFTS virus could interfere with IFN-associated pathway in the infected monocytes to impair their function of interferon production^8, 11^. Experiments performed on THP-1 monocytes showed that virus encoded NSs and N proteins could suppress interferon-β response by interfering IRF and NF-κB signaling to enhance viral replication, indicating that the inhibition of type I interferon response would contribute to the viral infection^8^. A study in Hela cells revealed that viral inclusion bodies (IBs) could sequester the viral RNA sensor, resulting in reduced downstream activation of TBK1/IKKε and IFN-β induction^9^. Nevertheless, direct evidence for the roles of innate immune response in pathogenesis and disease progress has not yet been established in SFTS patients. Especially the regulation of two important drivers of innate immune responses, plasmacytoid dendritic cells (pDCs) and myeloid dendritic cells (mDCs) which can rapidly produce large amount of type I interferon upon Toll like receptor (TLR) activation^12–14^ remains unknown although their roles during other viral infections, such as HIV, SIV, dengue virus and FMDV, had been well addressed previously^13, 15–17^.

In the current study, taking the advantage of a relatively large patient cohort (70 SFTS patients with various clinical manifestations), we conducted a comprehensive study focusing on the complex relationship among serum interferon level, viral load, pro-inflammatory cytokines and peripheral dendritic cells, and attempted to elucidate the roles of innate immunity and the regulatory network involving in SFTS virus infection.

## Results

### Clinical characteristics of SFTS patients showed dysfunction in hemostasis, coagulation and multi-organ injuries associated with the progress of SFTS

We divided 70 SFTS patients into three groups, as mild, severe and fatal groups based on both the severity and the outcome of the disease. Severe complications and their incidences in acute phase were shown in Table S1. The fatality of this cohort was 11.4%. The incidence of encephalitis and hemorrhage was the most and the second most predominant clinical manifestations of the infection, respectively. This phenomenon is coincident with clinical outcomes of other pathogenic phlebovirus infections, such as rift valley fever virus^18^. The median age of the patients in the severe and fatal groups were significantly higher than that of the mild patients, indicating that age is a risk factor of the disease, consistent with an earlier report^19^. A comprehensive set of clinical parameters was analyzed and summarized in Table S2. Although significantly declined, as compared to the normal range, WBC counts showed no statistic difference among the three groups.

Meanwhile, platelet counts exhibited significant differences between the mild and the severe groups. Indicators of organ injury, including ALT, LDH, CK and SCr, showed significant differences between the fatal and the other two groups. Therefore, we speculated that the impairment of hemostasis and coagulation function and multi-organ injuries were associated with the progress of SFTS.

### High serum viral load closely correlated with key clinical parameters

**Figure 1:**
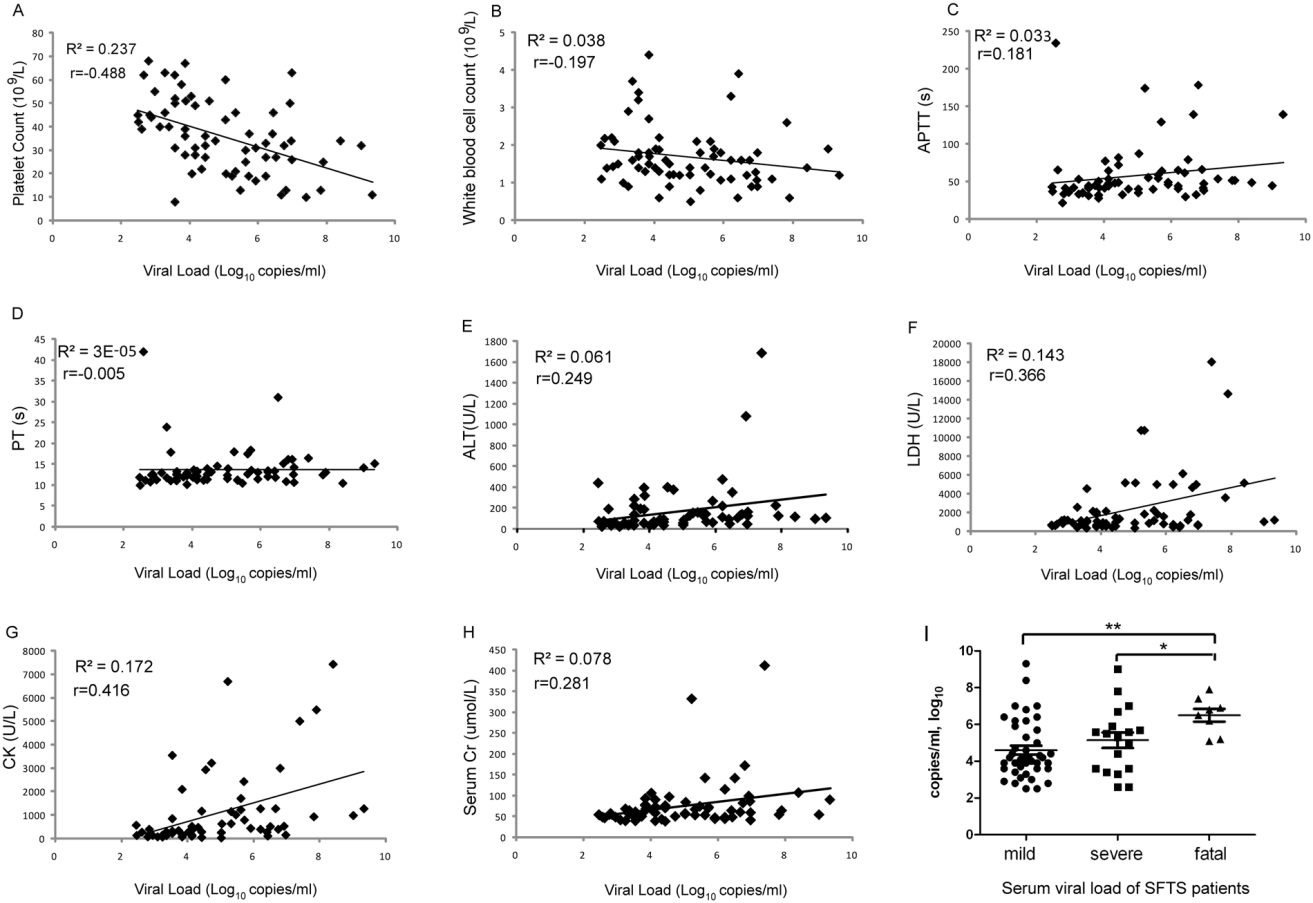
Correlation between serum viral load and clinical parameters in SFTS patients of various clinical severities. All serum samples were collected from the SFTS patients on the second day of their hospitalization. R^2^ represents coefficient of the determination. r represents Pearson correlation coefficient, with r values of 0–0.3, 0.3–0.5 and >0.5 indicating weak, moderate and strong correlation, respectively. (I) comparison of the serum viral load among three groups of SFTS patients. *p < 0.05, **p < 0.005.

The serum viral load in SFTS patients was investigated for its association with the pathogenesis and disease progress. The relationship between the serum viral load and a number of key clinical parameters was analyzed and presented in Fig. 1(A–H). In general, fatal patients (range 5.1–7.4, mean value 6.5) had significantly higher serum viral load than patients in both mild (range 2.5–9.3, mean value 4.6) and severe groups (range 2.6–7.8, mean value 5.1) as shown in Fig. 1(I). Platelet counts showed the strongest negative association with serum viral load (Fig. 1A). LDH and CK showed moderate positive associations with viral load. WBC, APTT, PT, ALT and SCr exhibited only weak associations with viral load. Together, these data provided evidence that high level of viral replication is associated with the reduction of platelets and multi-organ injuries, and acts as an independent risk factor for the aggravation of SFTS.

### Inhibition of IFN-β and elevation of pro-inflammatory cytokines in deceased SFTS patients

**Figure 2:**
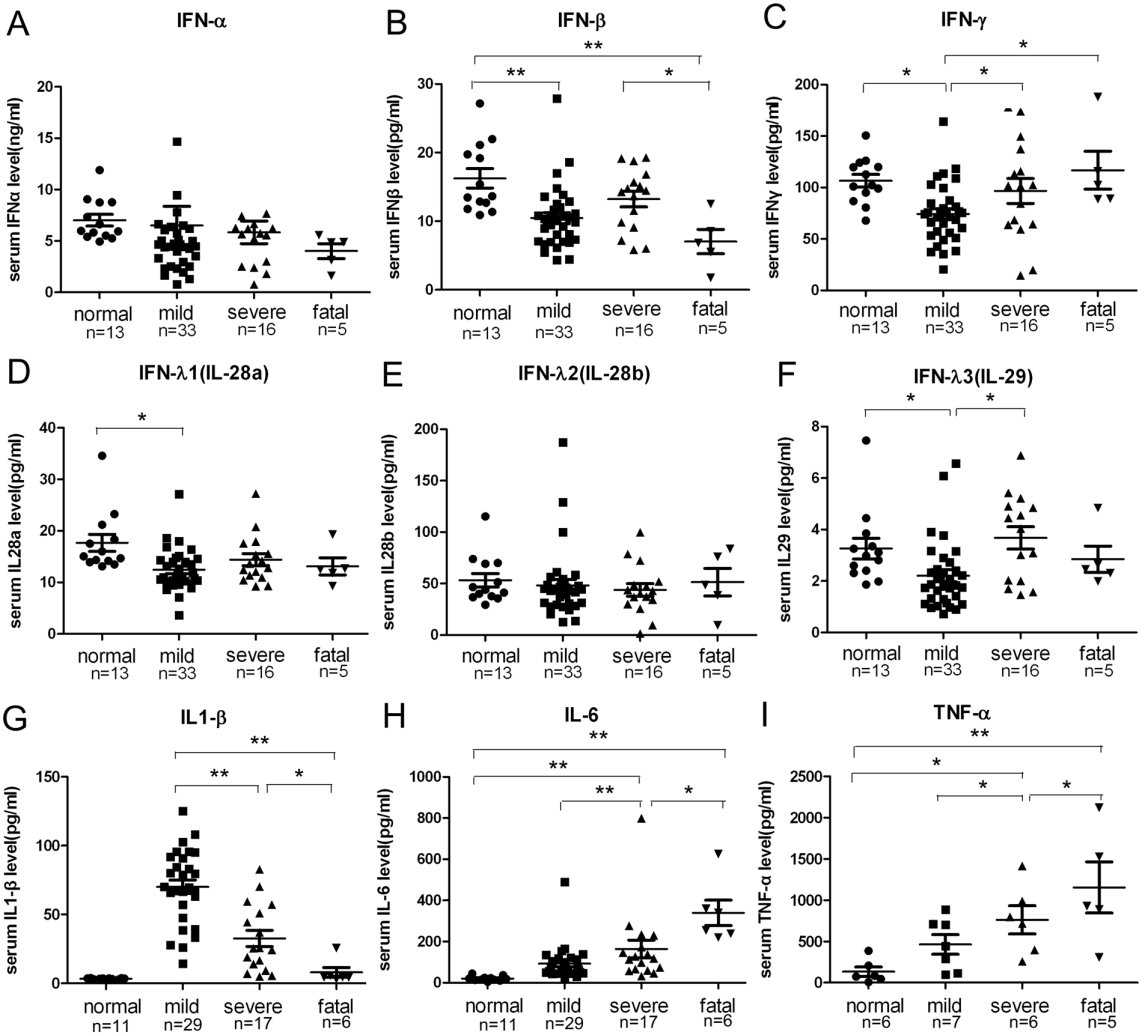
Comparison of type I, II and III Interferons and pro-inflammatory cytokines in the sera of SFTS patients of different clinical severities. IFN-α and IFN-β (**A,B**), IFN-γ (**C**), and IFNλ1-IFNλ3 (**D–F**) represent type I, type II and type III interferon response, respectively. (**G–I**) indicate serum level of three major pro-inflammatory cytokines IL-1β, IL-6, and TNF-α, respectively. All serum samples were collected from the patients on the second day of hospitalization. *p < 0.05, **p < 0.005.

Type I (IFN-α/β) and type II (IFN-γ) interferons serve as effectors of innate and adaptive immune response. The function of type III interferons, including IFN-λ1, IFN-λ2 and IFN-λ3, is considered to be analogous to that of type I IFNs^20^. Serum IFNs and three pro-inflammatory cytokines, IL-6, TNF-α and IL-1β, were measured during the early phase of acute SFTSV infection and shown in Fig. 2A–I. Serum IFN-α level exhibited no significant difference among three patient groups. Notably, serum IFN-β level was significantly reduced in the fatal patients, as compared to that in both healthy donors and mild patients (Fig. 2B). Serum IFN-γ level was the lowest in the mild group and elevated in the severe and fatal patients. Serum IFN-λ1 level in the mild group was lower than the healthy donors, but showed no statistical difference among the infected groups. Serum IFN-λ2 level had no difference in all the coupled groups. IFN-λ3 level was reduced in the mild group, when compared to the normal and the severe groups, but exhibited no significant difference among the severe, fatal and the normal groups. Three pro-inflammatory cytokines exhibited two diverse profiles. The serum IL-6 and TNF-α dramatically increased as the severity of the disease increased (Fig. 2H and I). In contrast, the serum IL-1β level was inversely correlated with the increase of the disease severity among three groups (Fig. 2G).

### Correlation between viral load, interferons and pro-inflammatory cytokines in acute SFTSV infection

**Figure 3:**
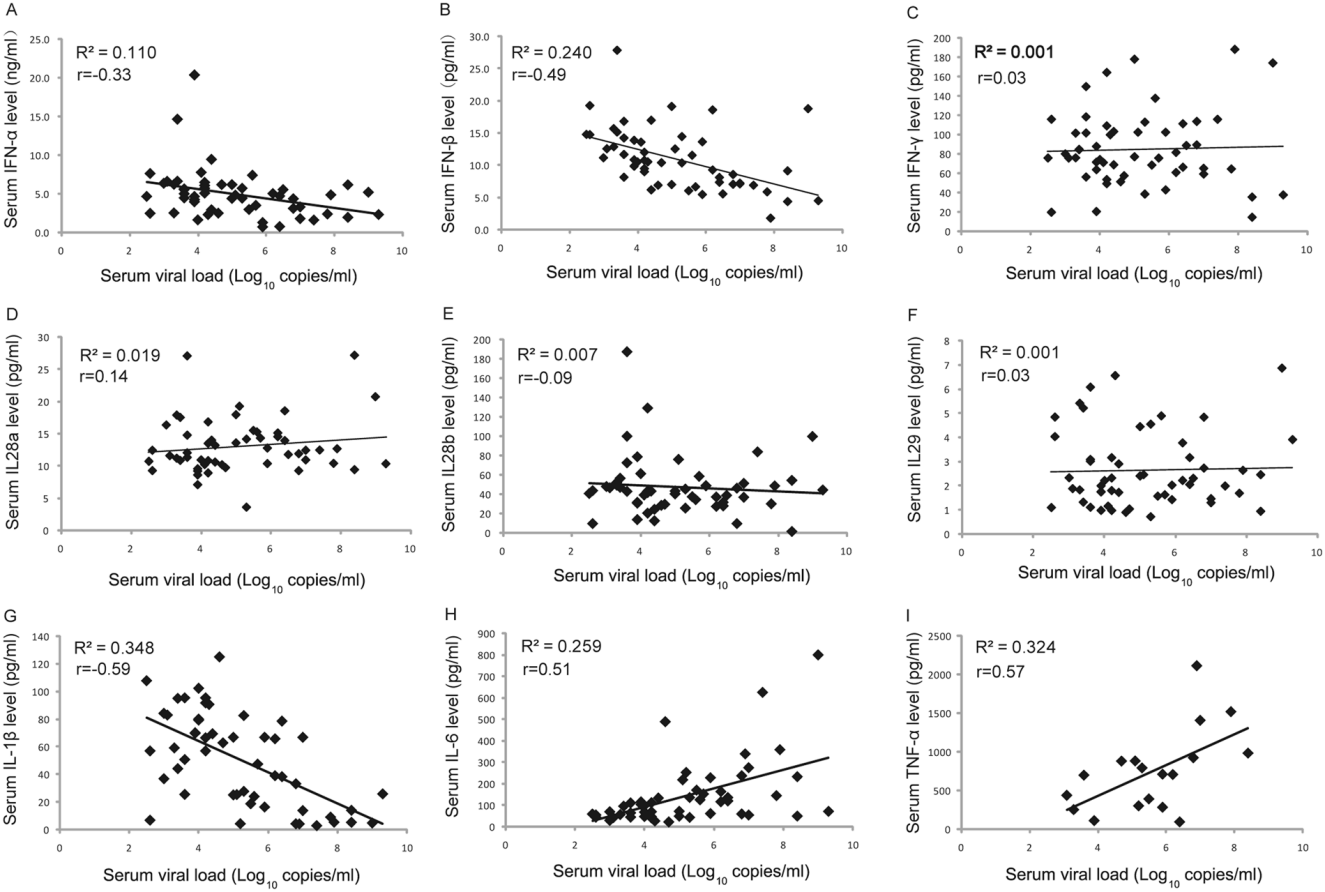
Correlation between serum viral load and serum levels of three types of Interferons and pro-inflammatory cytokines in the SFTS patients. All serum samples were collected from the SFTS patients on the second day of hospitalization. R^2^ represents coefficient of the determination and r represents Pearson correlation coefficient, with r value of 0–0.3, 0.3–0.5 and >0.5 indicating weak, moderate and strong correlation between the two variables, respectively.

We performed analysis on viral load, serum interferon level and proinflammatory cytokines to determine their relationships. Notably, serum IFN-β showed strong negative association with serum viral load (Fig. 3B). Interestingly, serum IFN-α level exhibited moderate negative correlation with serum viral load (Fig. 3A) although it did not vary significantly among three groups. Serum IFN-γ and type III IFNs showed weak associations with serum viral load (Fig. 3C–F). On the other hand, all pro-inflammatory cytokines, including IL-1β, IL-6 and TNF-α, manifested strong correlations with serum viral load (Fig. 3G–I). The data strongly suggest that uncontrolled viral replication contributed to the inhibition of IFN-β expression and promoted IL-6 and TNF-α expression, leading to cytokine storms.

### Transcriptional profile of immune related genes in peripheral monocytes of SFTS patients

To obtain an initial profile on the regulation of innate or adaptive immune related gene expression in peripheral blood, quantitative mRNA microarray analysis was performed in the monocytes and lymphocytes of three representative SFTS patients from the mild, severe and fatal groups, respectively. Since monocytes are considered as a major target for SFTSV, we carried out detailed analysis on the monocytes and presented the data in Figure S1. The rest of the data were summarized in Figures S2 and S3. Heatmap illustrates the expression of some key immune related genes in Figure S1A. We found that compared with other major pattern recognition receptors, mRNAs of TLR3 in the monocytes manifested the most significant modulation, and were down-regulated by 2.37, 4.94, and 9.5 log_2_ in the mild, severe and fatal patients, respectively, as the severity of the disease increases (Figure S1B). Additionally, interferon regulatory factors 3 and 7 (IRF3 and IRF7) exhibited similar regulatory patterns as TLR3. IFN-α1 mRNAs in monocytes were upregulated by 2.4 and 3.4 log_2_ in both mild and severe patients, respectively, but down-regulated in the fatal patient by 2.72 log_2_, as compared with the healthy donors (Figure S1B). IFN-β1 mRNAs was downregulated in the fatal patient by 5.77 log_2_, as compared with the healthy donors, but showed minimal changes in both mild and severe patients. Above data provided the clue that TLR3 pathway impairment might be the key cellular mechanism in the suppression of innate immune response. The mRNAs of IL-1β manifested dramatic downregulation in patients’ monocytes (−1.12, −5.02, −11.93 log_2_), respectively, in the mild, severe and fatal patients, consistently with its downregulated serum expression. Together, these observations suggest defective innate immune response to the SFTSV infection.

### Kinetics of myeloid and plasmacytoid dendritic cells in patients’ peripheral blood during acute SFTS virus infection

We analyzed the dynamic frequencies of circulating mDCs and pDCs of 13 survived and 7 deceased SFTS patients in the first three weeks post symptom onset using phenotypic markers previously reported. Because peripheral CD14^+^HLA-DR^+^ monocytes serve as the most abundant pool of APCs and precursors of dendritic cells, we compared their abundance as percentages of PBMCs among survived, deceased and healthy groups, and found no significant differences in the three-week period of follow-up (Data not shown). The frequencies of mDCs and pDCs were then analyzed for their percentages of total DCs and monocytes.

**Figure 4:**
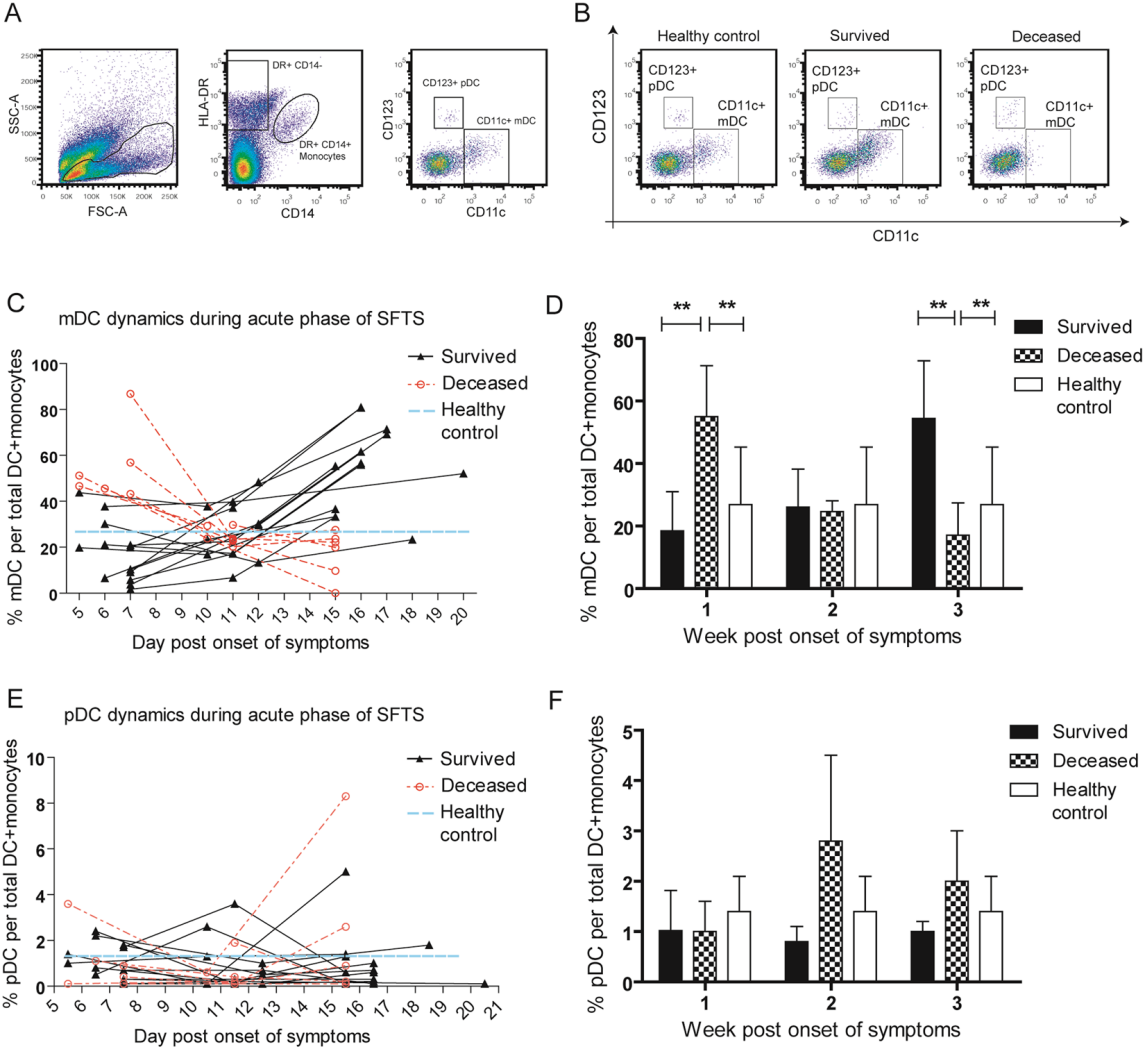
Dynamics of circulating myeloid dendritic cells (mDCs) and plasmacytoid dendritic cells (pDCs) during acute phase of SFTSV infected patients. The gating scheme used for identification of mDCs and pDCs in peripheral blood is shown in Fig. 4A. Three representative flow cytometry plots of mDCs and pDCs are from a survived and a deceased SFTS patients during acute phase as well as a healthy donor (Fig. 4B). Longitudinal assessments of percentage of circulating myeloid and plasmacytoid DCs in total DCs and monocytes from 13 survived and seven deceased patients during acute phase of SFTS are shown in Fig. 4C and E. Data of all day points are categorized into the first week (1~7^th^ day), the second week (8~14^th^ day) and the third week (15–21^th^ day) post onset of symptoms, and bar graphs (Fig. 4D and F) compare the mean ± SD of percentage of mDCs and pDCs in three different weeks. Percentage of circulating myeloid and plasmacytoid DCs in total DCs and monocytes from ten healthy donors were assessed as normal controls and were plotted in Fig. 4C and E as well as in Fig. 4D and F. *P < 0.05, **P < 0.005.

The response kinetics of CD11c^+^CD123^−^ mDCs and CD11c^−^CD123^+^ pDCs is shown in Fig. 4C and E. pDCs are normally gated using Lin- and BDCA2+ markers, whereas a brief gating as CD11c−CD123+ (pDCs) was used in the present study. We observed the distinct fluctuating profiles of mDCs in survived and deceased patients. mDCs of the deceased group had significantly higher percentages as compared to the survived (p = 0.002) and healthy groups (p = 0.000) in the first week, and manifested a decline in the following week and the third week (Fig. 4D). In the third week post symptom onset, the percentage of mDCs of the deceased group was significantly lower than the survived (p = 0.000) and healthy groups (p = 0.002), and 6 out of 7 patients succumbed to the infection in this week. By contrast, all survived patients manifested steady increase of the CD11c^+^CD123^−^ population, suggesting a robust mDC up-regulation in response to SFTSV infection. Meanwhile, the variation of CD11c^−^CD123^+^ pDC population appeared to be sporadic and showed no significant differences among three groups and no significant differences for the period of the analysis.

### Dynamic analysis of TLR3 expression by peripheral mDCs, pDCs and CD14^+^HLA-DR^+^ monocytes

**Figure 5:**
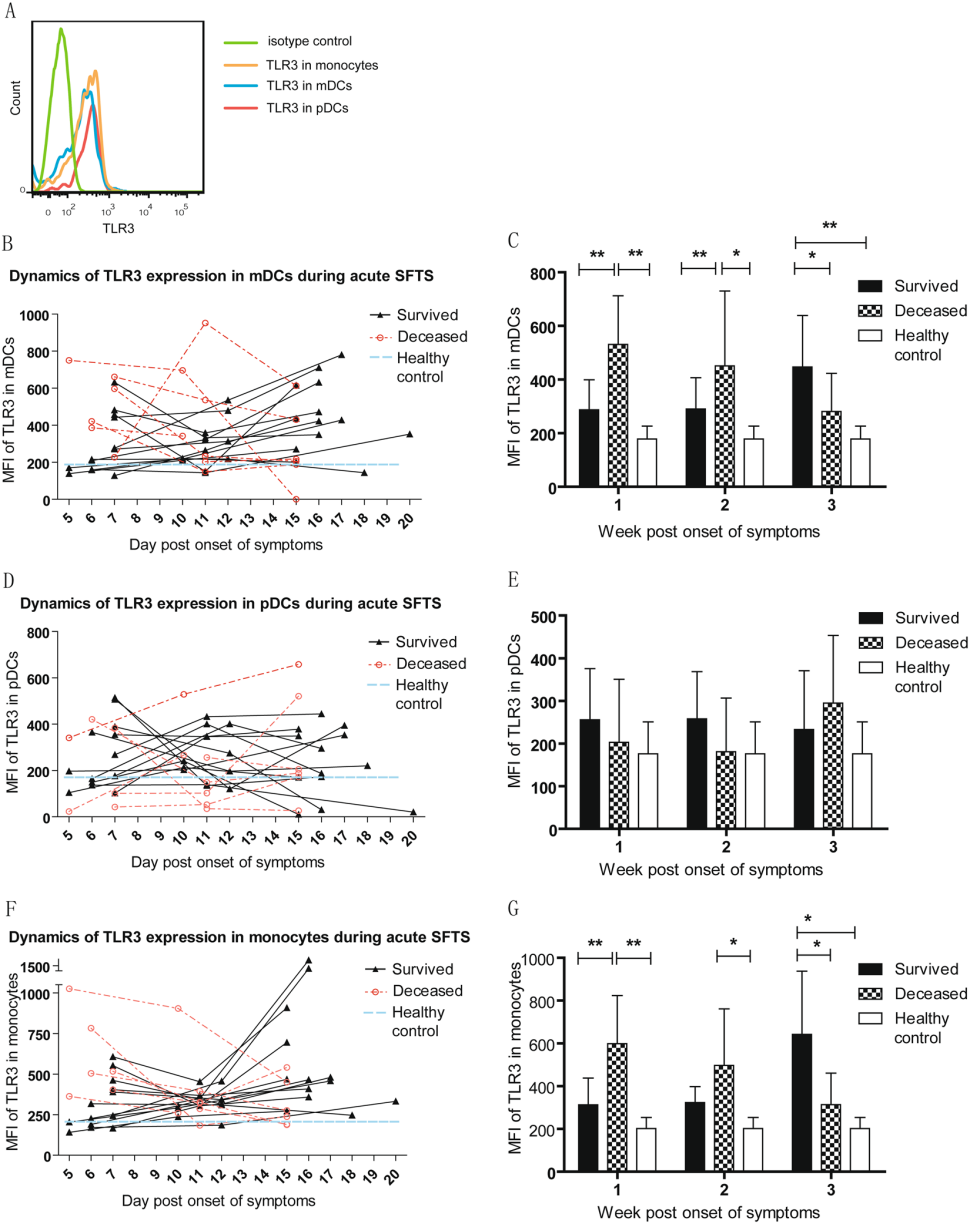
Dynamics of Toll like receptor 3 (TLR3) expression by circulating mDCs, pDCs and CD14+HLA-DR+ monocytes of SFTSV infected patients during acute phase. Representative histograms of TLR3 expression by mDCs, pDCs and monocytesare shown in Fig. 5A. Dynamics of TLR3 expressionby mDCs, pDCs and monocytes from 13 survived and seven deceased patients during acute phase of SFTS, measured by mean fluorescence intensity (MFI), are shown in Fig. 5B,D and F. Mean Fluorescence Intensity (MFI) of intracellular TLR3 expression by the cells of all day points are categorized into the first week (1~7^th^ day), the second week (8~14^th^ day) and the third week (15–21^th^ day) post onset of symptoms, and bar graphs (Fig. 5C,E and G) compare the mean ± SD of MFI of TLR3 by mDCs, pDCs and monocytes in three different weeks. TLR3 expression by mDCs, pDCs and monocytes fromten healthy donors, measured by mean fluorescence intensity (MFI), were assessed as normal controls and plotted in Fig. 5B,D and F as well as in Fig. 5C,E and G. *P < 0.05, **P < 0.005.

TLR3 expression was identified based on the mDCs, pDCs and monocytes gate using intracellular staining, as shown in Fig. 4A, and measured as MFI which was calculated by subtracting the MFI of the corresponding isotype control (Fig. 5A). The dynamic TLR3 expression of three patient groups for the three-week period post symptom onset was presented in Fig. 5B–G. Our results demonstrated that the TLR3 expressions by mDCs (Fig. 5B) and monocytes (Fig. 5F) in deceased group were gradually downregulated during the acute phase, and significantly declined as compared with the survived group at the third week (p = 0.048, 0.04 respectively), although TLR3 expression in the deceased group was significantly higher than the survived group at the first week (p = 0.003, 0.002, respectively). On the contrary, the expression of TLR3 in both mDCs and monocytes in the survived group increased for the three-week duration of infection and reached peak levels in the third week after symptom onset, indicating that TLR3 upregulation in mDCs and their precursors play positive roles in the convalescence of SFTS disease. The same analysis was conducted on pDCs population (Fig. 5D); however, we observed no statistically significant differences of TLR3 expression among the three groups for the three-week period post symptom onset.

## Discussion

SFTS, an emerging viral hemorrhagic fever with high fatality rates at 12–30%^1–3^, imposes great challenge to the public health in the East Asian region. No effective vaccine is available yet and clinical interventions are limited. Limited studies reported clinical manifestation of the disease and a number of independent risk factors of prognosis, including age and high level of viremia^3, 6, 19^. In the present study, we observed that the reduction of platelets is strongly correlated with the severity of SFTS and viremia level, indicating that the platelet count is a prognostic marker for the SFTS patients. Based on our clinical data sets, we first analyzed all three important indicators of coagulation function, including APTT, PT, and TT, representing intrinsic, extrinsic, and final coagulation pathway, respectively. Thrombin Time (TT) of six fatal patients who suffered lethal hemorrhage was beyond the detection limit, but no statistical significance of prolongation of both APTT and PT in the fatal group was observed, as compared to the severe group. This non-parallel phenomenon between APTT/PT and TT was first observed in SFTS patients in our study, although similar phenomenon has been observed in HSV infection and was attributed to the virally encoded glycoprotein C that enhanced activation of FX^21^.

To elucidate the status of innate immune response and the regulatory mechanisms, as well as their roles in the pathogenesis of SFTS, we performed a comprehensive analysis on serum IFN level, viral load, key cytokines involved in the innate immunity, and dynamics of major dendritic cell populations during acute SFTS infection in the patient cohort. Initially, two distinct families of interferons (IFNs), type I (IFN-α/β) and type III (IFN-λ) IFNs, both of which engage in innate immunity, were determined in the sera of 59 patients during the first week post symptom onset, and serum IFNβ was found to exhibit a statistically significant reduction in the fatal patients while serum IFN-α was slightly inhibited. A recent study^22^ reported that IFNa increased with disease severity in SFTS infection. The discrepancy may be caused by the differences of sampling time between our and Liu’s studies. The samples in our study were collected in the first week post disease onset whereas samples in Liu’s study were collected within 2 weeks since onset. Therefore, our results represent serum IFN-α level at an earlier phase of acute SFTS infection. Although their regulations are similar, IFN-β and IFN-α are secreted by different cells or regulated differently^23, 24^. Plasmacytoid and conventional DCs secrete high levels of IFN-α, whereas fibroblasts secrete mainly IFN-β as an initial response to infection but switch to IFN-α during the subsequent amplification phase of the IFN response^25^. The regulatory mechanism may be infection related. A report showed that p53 pathway was more strongly activated by IFN-β than by IFN-α in HTLV infection. Additionally, IFN-β upregulated NF-κB activation, whereas IFN-α modestly downregulated NF-κB pathway activation^26^.

Serum viral loads of these patients at the same time point post symptom onset were quantitated. Correlation analysis showed that high viremia had tight association with suppressed IFN-β production. Considering that type I interferons (IFNα/β) are mainly produced and secreted by dendritic cells, we conducted further investigation of the DCs in the peripheral blood of patients. Dendritic cells (DCs) play crucial roles in innate immunity to eliminate viral infection as the first line of host defense. Peripheral DCs include two major populations: pDCs and mDCs, which are phenotypically defined as lineage (Lin)- HLA-DR^+^CD123^+^CD11c^−^ and Lin-HLA-DR^+^ CD11c^+^ CD123^−^, respectively^15, 27^. Human mDC population, known as classical DCs, is identified to be homogeneous and contains two non-overlapping subsets: CD11c^+^ (BDCA-1) and CD141^+^ (BDCA-3) mDCs^14, 15^. Both pDCs and mDCs express an important pattern recognition receptor, TLR3, and rapidly produce large amount of type I interferons when activated by double-stranded RNA (dsRNA) during viral replication^14, 28^. The roles of pDCs and mDCs have been well established in some chronic and acute viral infections. The parallel loss of both pDCs and mDCs from blood and lymphoid tissue has been observed to be associated with progressive disease in HIV-infected individuals^29^. Among acute viral infections, high titer influenza A (H1N1) viral infection can stimulate rapid pDC apoptosis^30^. Dengue virus has been shown to infect dendritic cells and their precursors using DC-SIGN as a receptor and affect their differentiation and function^16^. Recent evidence has also shown that the interaction of SFTSV glycoproteins (Gn/Gc) with DC-SIGN facilitates the viral entry into target cells, including dendritic cells and monocytes^31, 32^. However, the quantitative changes and functions of mDCs and pDCs along the disease course and their associations with the progress of SFTS in patients are not yet known.

In the present study, the kinetics of the frequencies of peripheral mDCs and pDCs during first three weeks post symptom onset were measured using multi-color flow cytometry in both the survived and deceased groups, as well as 10 healthy donors serving as normal controls. Strikingly, although mDC population of the deceased group constituted between 43.2–86.8% of total DCs plus CD14^+^HLA-DR^+^ monocytes at the first week, as compared with 3.7–43.8% in the survived group and 26.9% in the healthy donors, serum IFN-β level of the fatal patients during the same period of time was significantly lower than the other two groups. This phenomenon strongly indicated that mDC increase in the deceased patients at the early stage of infection was not accompanied with the enhancement of function. We observed in the same patient cohort that both CD80 and CD86 expressions in mDCs were drastically inhibited in the three-week duration in the deceased group but not in the survived patients, suggesting functional defects in mDCs (manuscript in preparation, Song *et al*.). The dynamic changes in the frequency of mDCs indicate that the down-regulation of mDC frequency in the deceased group is associated with the aggravation of SFTS. Based upon above observations, we postulate that the differentiation of mDC was activated following initial SFTSV infection, but quickly mitigated by high titers of virus and inflammatory cytokines such as TNF-α and IL-6. On the contrary, the frequencies of mDCs in the survived patients manifested steady increases with the relieved clinical symptoms and reached the peak at the third week of convalescence. Despite previous research indicating that circulating pDCs produced more IFN-I than any other cell types in the blood^13^ and their change in number and function played important roles in HIV and Influenza A infection^29, 30^, the frequency of circulating pDCs during acute SFTSV infection exhibited no significant variations among the survived, deceased and healthy groups. It is plausible to suggest that the effective differentiation of circulating mDCs, but not pDCs, play positive roles in the control of SFTSV infection.

In order to better understand the roles of cytokines engaging in the immune activation and innate immunity in SFTS, we analyzed three representative cytokines in the present study, including TNF-α, IL-6 and IL-1β. As the major pro-inflammatory cytokines in virus infection^33, 34^, serum TNF-α and IL-6 are good indicators of cytokine storm. Additionally, IL-1β, a pleiotropic cytokine^35^ involved in both inflammation and innate immune response, was also determined. Our observations revealed that both TNF-α and IL-6 exhibited coherent upregulation and increased with the elevation of disease severity. Contrary to the modulation of IL-6 and TNF-α, the serum IL-1β level exhibited strong negative association with the viral load and severity of disease, implicating the protective roles of IL-1β against SFTSV infection in acute phase. In fact, as an important component of innate immune response, IL-1β is secreted by monocyte-derived dendritic cells and macrophages and involved in a variety of cellular activities and antiviral activity^36–38^. The down modulation of IL-1β is a further evidence of mDC dysfunction during the infection. In addition, the downregulation of IFN-β will lead to inhibition of IL-1β secretion via the type I IFN positive feedback loop^38^, consistent with our data indicating that both IFN-β and IL-1β were inhibited in the early stage of fatal SFTSV infection. Interestingly, the changing profile of serum IL-1β in current study differed from the report by Sun *et al*. In that study, serum IL-1β of non-fatal group was significantly lower than fatal group in the acute phase of SFTS, but dramatically elevated in 50–150 days after onset of illness, exhibiting late IL-1β response in the convalescent phase of SFTS. The differences could be due to the patient cohorts used in the studies^39^ or the viruses that may have different pathogenic properties.

To investigate the regulatory mechanisms in cells involved in innate immunity, we assessed the gene expression in the peripheral monocytes of three representative patients and one healthy donor using a Qiagen microarray panel containing 84 pre-selected genes engaging in innate and adaptive immune response (Figure S2). The reasons for choosing peripheral monocytes include their relatively large population in patients’ blood so as to extract the desirable amount of RNA for detection, and that they are the major precursors of peripheral DCs and the major cellular target of SFTSV as previously reported. Among two major families of pattern recognition receptors, including TLRs (TLR1-TLR9) and RIG-I (DDX58), we found that only the expression of TLR3, except TLR5, manifested significant change in patients and down-regulated by nearly 10 folds in the fatal patient, as compared with the normal control. Besides, two specific adaptors in TLR3 signaling pathway, IRF3 and IRF7^40^, and the effectors IFN-α1 and IFN-β1 all exhibited down-regulated expressions with the increase of SFTS severity. Considering TLR5 plays a feeble role in virus infection, it was not discussed in the present study. Additionally, the IL-1β mRNA level in monocytes decreased as the severity of the disease increased, consistent with the changing profile in serum. Based on the above observations, we postulate that SFTSV infection downregulates TLR3 expression, resulting in the dysfunction of innate immune response to SFTSV infection.

In the measurement of TLR3 expression using multi-color flow cytometry, we found that the changing profiles of monocytes and mDCs exhibited high similarity as evidenced by the kinetics of TLR3 expression in CD14^+^HLA-DR^+^ monocytes and mDCs. TLR3 expression in both cell types exhibited gradual improvement with the convalescence of SFTS in the survived group, but decreased in the deceased group with the aggravation of the disease. The regulation of TLR3 expression in mDCs is highly consistent with the mDCs frequency in the peripheral blood of SFTS patients. This result indicates that not only the number, but also the function of circulating mDCs is greatly improved in those patients who overcome the SFTSV infection. Interestingly, in the survived patients, the higher mDC frequency was proportional to the TLR3 expression in CD14^+^HLA-DR^+^ monocytes, which is consistent with a previously published report that DC maturation depends on the signaling in TLR3 pathway^41^. It is plausible to suggest that virus-mediated down-regulation of TLR3 in CD14^+^HLA-DR^+^ monocytes result in repressed mDC differentiation, leading to the defective antiviral response.

In summary, the current study provides clinical evidence suggesting that dysfunction of innate immune response and cytokine storm play important roles in the pathogenesis of SFTS. Our longitudinal analysis on the dendritic cell kinetics illustrates that effective differentiation of mDCs, but not pDCs, plays critical roles in the convalescence of SFTS patients. Importantly, among the major pattern recognition receptors, the regulation of TLR3 and its signaling pathway could be the key regulatory mechanism in SFTSV-induced innate immunity.

## Materials and Methods

### Patient cohort

Informed consent was obtained from all subjects, in accordance with the Declaration of Helsinki, and the research was approved by the Ethics Committee of Nanjing Drum Tower Hospital. All experiments were performed in accordance with relevant guidelines and regulations. During 2011 to 2016, 70 SFTS patients were admitted in Nanjing Drum Tower Hospital and were confirmed of SFTS virus infection by quantitative RT-PCR. Severity assessment was conducted based on the clinical manifestations and the clinical outcome of the patients, particularly the occurring of severe complications as encephalitis, hemorrhage, and multi-organ failure, in according to the clinical criterion of acute infectious diseases from Chinese Medical Association.

### ELISA

Serum levels of IFN-α, IFN-β, IFN-ϒ, IL-28A, IL-28B and IL-29 in healthy donors and patients were determined by commercial enzyme-linked immunosorbent assay (ELISA) kits from eBioscience Company (San Diego, USA), and IL-1β, IL-6, and TNF-α were measured with kits from Raybiotech (Norcross GA, USA). The levels of all above cytokines were determined during the first week after onset of symptoms.

### Quantitative RT-PCR

Patients’ blood samples for viral quantitative PCR were collected during the first week post onset of symptoms. The viral load of SFTSV in patients’ serum was determined by the Laboratory of Jiangsu and Nanjing CDC. The primer set was as follow: forward primer: 5′-GGGTCCCTGAAGGAGTTGTAAA-3′, reverse primer: 5′-TGCCTTCACCAAGACTATCAATGT-3′, probe: 5′-FAM-TTCTGTCTTGCTGGCTCCGCGC-BHQ2-3′.

### Peripheral blood mononuclear cells (PBMCs) isolation

PBMCs were isolated by density gradient centrifugation using Ficoll-Paque Plus (GE Health Bioscience, Sweden) according to the manufacture’s protocol. PBMCs were incubated in plate for 3 h at 37 °C in RPMI-1640 supplemented with 10% heat inactivated FBS. Adherent and suspension cells in the PBMCs were collected as monocytes and lymphocytes.

### RNA extraction

Total RNA was extracted from lymphocytes and monocytes of healthy donors or patients using RNeasy Micro Kit (QIAgen) according to the manufacturer’s protocol. RNA quantity was determined by spectrophotometry using a Nanodrop 1000 (Thermo Fisher).

### Innate and adaptive immune responses RT^2^ Profiler PCR Array

The RT^2^ Profiler PCR Array on the total RNA from monocytes or lymphocytes was performed by Qiagen China. The mRNA transcription levels of 84 innate and adaptive immune responses were quantitatively determined. Results of the RT^2^ Profiler PCR Array were analyzed by Qiagen China, while ACTB and GAPHD were set as house-keeping gene control in the measurement and analysis.

### Cell staining and flow cytometry

Peripheral blood white cells from SFTS patients were isolated by using BD Pharm Lyse™ (BD Pharmingen, USA). The patient’s white cells were stored in liquid nitrogen before use. PE-cy7 conjugated anti-human HLA-DR, FITC conjugated anti-human CD11c, APC conjugated anti-human CD123 and APC-cy7 conjugated anti-human CD14 antibodies (BD Pharmingen, USA) were used in surface staining to determine dendritic cells and monocytes according to manufacturer’s protocols, whereas intracellular TLR3 expression was determined by using PE-conjugated anti-human TLR3 antibodies (eBioscience, USA) according to manufacturer’s protocols. Isotype control antibodies (BD Pharmingen, USA & eBioscience, USA) were used to determine the baseline stain. Stained cells were measured by BD FACS Aria II (BD Biosciences, China) and the results were analyzed by FlowJo™ (Flowjo LLC, USA).

### Statistical analysis

Continuous variables were reported as means ± standard deviations (SD). Data were analyzed by SPSS 17.0 software. Group means were compared using ANOVA. Pearson’s correlation tests were used to measure the strength of association between variables.

## Electronic supplementary material

Supplementary Figures and Tables

## References

[1] Yu XJ (2011). Fever with thrombocytopenia associated with a novel bunyavirus in China. N Engl J Med.

[2] Zhang YZ (2012). Hemorrhagic fever caused by a novel Bunyavirus in China: pathogenesis and correlates of fatal outcome. Clin Infect Dis.

[3] Li J (2014). Concurrent measurement of dynamic changes in viral load, serum enzymes, T cell subsets, and cytokines in patients with severe fever with thrombocytopenia syndrome. PLoS One.

[4] Shin J, Kwon D, Youn SK, Park JH (2015). Characteristics and Factors Associated with Death among Patients Hospitalized for Severe Fever with Thrombocytopenia Syndrome, South Korea, 2013. Emerg Infect Dis.

[5] Yoshikawa T (2015). Phylogenetic and Geographic Relationships of Severe Fever With Thrombocytopenia Syndrome Virus in China, South Korea, and Japan. J Infect Dis.

[6] Sun Y (2012). Host cytokine storm is associated with disease severity of severe fever with thrombocytopenia syndrome. J Infect Dis.

[7] Deng B (2012). Cytokine and chemokine levels in patients with severe fever with thrombocytopenia syndrome virus. PLoS One.

[8] Qu B (2012). Suppression of the interferon and NF-kappaB responses by severe fever with thrombocytopenia syndrome virus. J Virol.

[9] Wu X (2014). Evasion of antiviral immunity through sequestering of TBK1/IKKepsilon/IRF3 into viral inclusion bodies. J Virol.

[10] Jin C (2012). Pathogenesis of emerging severe fever with thrombocytopenia syndrome virus in C57/BL6 mouse model. Proc Natl Acad Sci USA.

[11] Wu X (2014). Roles of viroplasm-like structures formed by nonstructural protein NSs in infection with severe fever with thrombocytopenia syndrome virus. FASEB J.

[12] Menon M, Blair PA, Isenberg DA, Mauri C (2016). A Regulatory Feedback between Plasmacytoid Dendritic Cells and Regulatory B Cells Is Aberrant in Systemic Lupus Erythematosus. Immunity.

[13] Royle CM, Graham DR, Sharma S, Fuchs D, Boasso A (2014). HIV-1 and HIV-2 differentially mature plasmacytoid dendritic cells into IFN-producing cells or APCs. Journal of immunology.

[14] Yu CI (2014). Human CD141+ dendritic cells induce CD4+ T cells to produce type 2 cytokines. Journal of immunology.

[15] Soulas C (2015). Distinct phenotype, longitudinal changes of numbers and cell-associated virus in blood dendritic cells in SIV-infected CD8-lymphocyte depleted macaques. PLoS One.

[16] Dejnirattisai W (2008). A complex interplay among virus, dendritic cells, T cells, and cytokines in dengue virus infections. Journal of immunology.

[17] Sei JJ, Waters RA, Kenney M, Barlow JW, Golde WT (2016). Effect of Foot-and-Mouth Disease Virus Infection on the Frequency, Phenotype and Function of Circulating Dendritic Cells in Cattle. PLoS One.

[18] Boushab BM (2016). Severe Human Illness Caused by Rift Valley Fever Virus in Mauritania, 2015. Open forum infectious diseases.

[19] Ding S (2014). Age is a critical risk factor for severe fever with thrombocytopenia syndrome. PLoS One.

[20] Lazear HM, Nice TJ, Diamond MS (2015). Interferon-lambda: Immune Functions at Barrier Surfaces and Beyond. Immunity.

[21] Antoniak S, Mackman N (2014). Multiple roles of the coagulation protease cascade during virus infection. Blood.

[22] Liu MM, Lei XY, Yu H, Zhang JZ, Yu XJ (2017). Correlation of cytokine level with the severity of severe fever with thrombocytopenia syndrome. Virology journal.

[23] Platanias LC (2005). Mechanisms of type-I- and type-II-interferon-mediated signalling. Nature reviews. Immunology.

[24] Darnell JE, Kerr IM, Stark GR (1994). Jak-STAT pathways and transcriptional activation in response to IFNs and other extracellular signaling proteins. Science.

[25] Weber F, Kochs G, Haller O (2004). Inverse interference: how viruses fight the interferon system. Viral immunology.

[26] Dierckx T (2017). IFN-beta induces greater antiproliferative and proapoptotic effects and increased p53 signaling compared with IFN-alpha in PBMCs of Adult T-cell Leukemia/Lymphoma patients. Blood cancer journal.

[27] Deal EM, Lahl K, Narvaez CF, Butcher EC, Greenberg HB (2013). Plasmacytoid dendritic cells promote rotavirus-induced human and murine B cell responses. The Journal of clinical investigation.

[28] Yang JY (2016). Enteric Viruses Ameliorate Gut Inflammation via Toll-like Receptor 3 and Toll-like Receptor 7-Mediated Interferon-beta Production. Immunity.

[29] Brown KN, Trichel A, Barratt-Boyes SM (2007). Parallel loss of myeloid and plasmacytoid dendritic cells from blood and lymphoid tissue in simian AIDS. Journal of immunology.

[30] Thomas JM (2014). Differential responses of plasmacytoid dendritic cells to influenza virus and distinct viral pathogens. J Virol.

[31] Hofmann H (2013). Severe fever with thrombocytopenia virus glycoproteins are targeted by neutralizing antibodies and can use DC-SIGN as a receptor for pH-dependent entry into human and animal cell lines. J Virol.

[32] Tani H (2016). Characterization of Glycoprotein-Mediated Entry of Severe Fever with Thrombocytopenia Syndrome Virus. J Virol.

[33] Bryant-Hudson KM, Gurung HR, Zheng M, Carr DJ (2014). Tumor necrosis factor alpha and interleukin-6 facilitate corneal lymphangiogenesis in response to herpes simplex virus 1 infection. Journal of virology.

[34] To KK (2016). Human H7N9 virus induces a more pronounced pro-inflammatory cytokine but an attenuated interferon response in human bronchial epithelial cells when compared with an epidemiologically-linked chicken H7N9 virus. Virology journal.

[35] Jalbert E (2013). IL-1Beta enriched monocytes mount massive IL-6 responses to common inflammatory triggers among chronically HIV-1 infected adults on stable anti-retroviral therapy at risk for cardiovascular disease. PloS one.

[36] Barjesteh N (2014). TLR ligands induce antiviral responses in chicken macrophages. PloS one.

[37] Vlahos R (2011). Inhibition of Nox2 oxidase activity ameliorates influenza A virus-induced lung inflammation. PLoS pathogens.

[38] Pothlichet J (2013). Type I IFN triggers RIG-I/TLR3/NLRP3-dependent inflammasome activation in influenza A virus infected cells. PLoS pathogens.

[39] Ding SJ (2016). Correlation Between HLA-A, B and DRB1 Alleles and Severe Fever with Thrombocytopenia Syndrome. PLoS Negl Trop Dis.

[40] Sehgal R (2015). Impaired monocyte-macrophage functions and defective Toll-like receptor signaling in hepatitis E virus-infected pregnant women with acute liver failure. Hepatology.

[41] Hu W (2015). Differential outcome of TRIF-mediated signaling in TLR4 and TLR3 induced DC maturation. Proc Natl Acad Sci USA.

