# Supplementary material for: Downregulation of Interferon-β and Inhibition of TLR3 Expression are associated with Fatal Outcome of Severe Fever with Thrombocytopenia Syndrome
Source: Sci Rep. 2017 Jul 26;7:6532. doi: 10.1038/s41598-017-06921-6 (PMC5529500; doi:10.1038/s41598-017-06921-6)
Supplement: Supplementary file 1 — Supplementary Figures and Tables [file 41598_2017_6921_MOESM1_ESM.doc]

**Supplementary Information**

Downregulation of Interferon-β and Inhibition of TLR3 Expression are associated with Fatal Outcome of Severe Fever with Thrombocytopenia Syndrome

Peixin Song^1#^, Nan Zheng^2,3#^, Li Zhang^3^, Yong Liu^4^,Taoyu Chen^3^, Changjun Bao^5^, Zhifeng Li^5^, Wei Yong^6^, Yongyang Zhang^1^, Chao Wu^1^, Zhiwei Wu^2,3*^

*^1^Department of Infectious Diseases, Nanjing Drum Tower Hospital, Nanjing University Medical School.*

*^2^State Key Lab of Analytical Chemistry for Life Science, Nanjing University, Nanjing, PR China.*

*^3^Center for Public Health Research, Nanjing University Medical School, Nanjing, PR China.*

*^4^Department of Experimental Medicine, Nanjing Drum Tower Hospital, Nanjing University Medical School.*

*^5^Jiangsu Provincial Center for Disease Control and Prevention, PR China.*

*^6^Nanjing Center for Disease Control and Prevention, PR China.*

#These authors contributed equally to the article

*Corresponding author: Z. Wu, Mailing address: Center for Public Health Research, Nanjing University Medical School, 22# Hankou Road, Nanjing, Jiangsu Province, 210093, China. Phone: 86 (25) 8368-6092. Fax: 86 (25) 8359-6023.

E-mail address: [wzhw@nju.edu.cn](mailto:wzhw@nju.edu.cn).

**Supplementary Figure Legends**

**Figure S1. Modulation of immune-related gene expression in monocytes.** Three patient’s samples were collected at Day 12 (mild), Day 14 (severe), and Day 15 (fatal) post onset of symptoms for quantitative microarray analysis. Their total mRNAs in peripheral monocytes were extracted and quantified by Profiler PCR array using an Innate & Adaptive Immune Responses chip. Heatmap shows the expression of some key immune related genes in monocytes compared to the healthy individual control (Figure S1A). The changing magnitude of these genes are displayed in Figure S1B.

**Figure S2. Measurement of relative expression of 84 genes involved in innate and adaptive immune response in peripheral monocytes of SFTS patients during acute phase.** Total mRNAs in peripheral monocytes of three representative SFTS patients were extracted and quantified by Profiler PCR array using an Innate & Adaptive Immune Responses chip. The regulation of gene expression was measured in fold change (Log_2_) compared with normal control.

**Figure S3. Measurement of relative expression of 84 genes involved in innate and adaptive immune response in peripheral lymphocytes of SFTS patients during acute phase.** Total mRNAs in peripheral lymphocytes of three representative SFTS patients were extracted and quantified by Profiler PCR array using an Innate & Adaptive Immune Responses chip. The regulation of gene expression was measured in fold change (Log_2_) compared with normal control.

**Figure S4. The frequency of mDC and pDC before and after G-CSF administration.**

**Supplementary Figures**


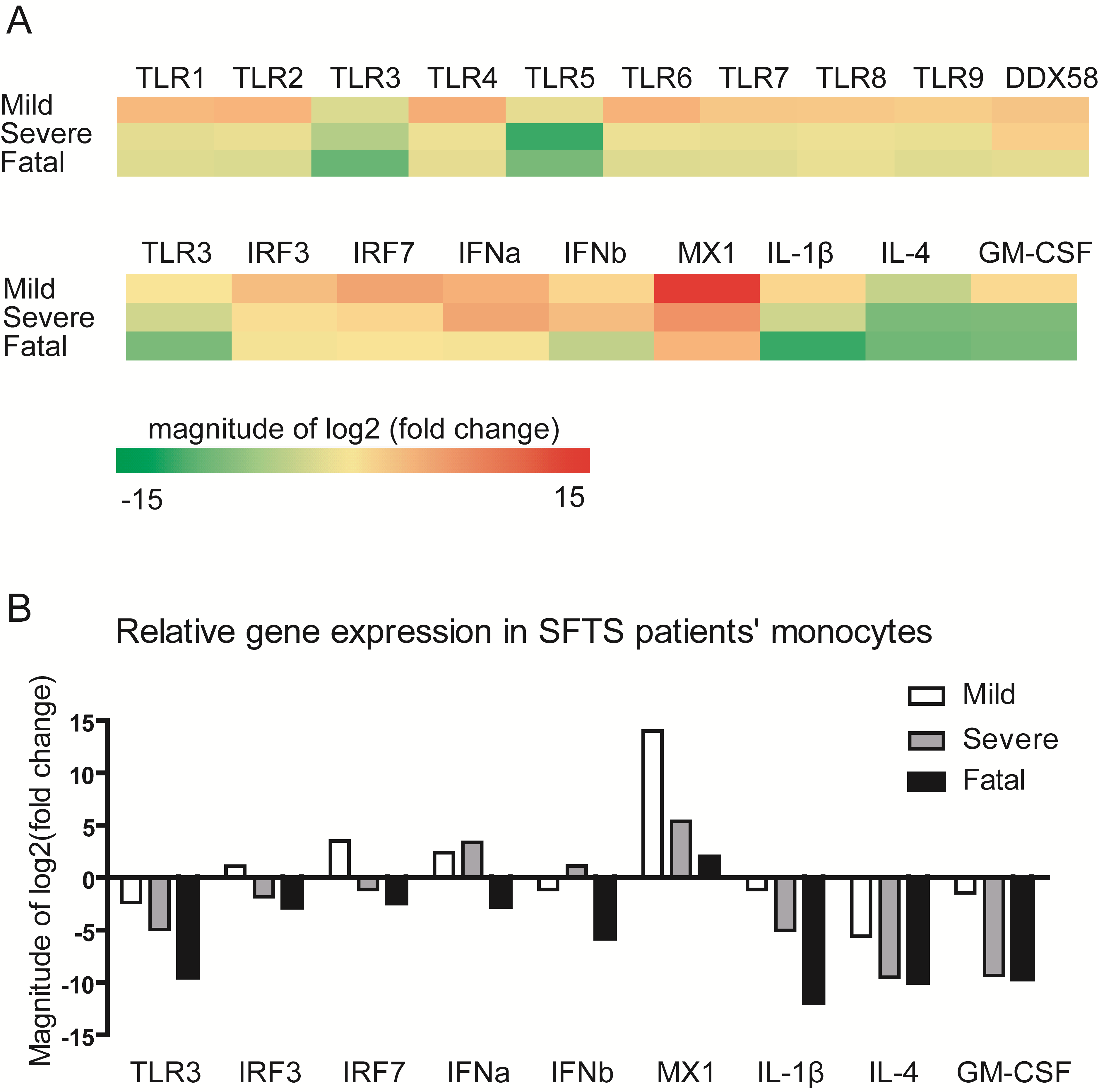


**Figure S1. Modulation of immune-related gene expression in monocytes.**


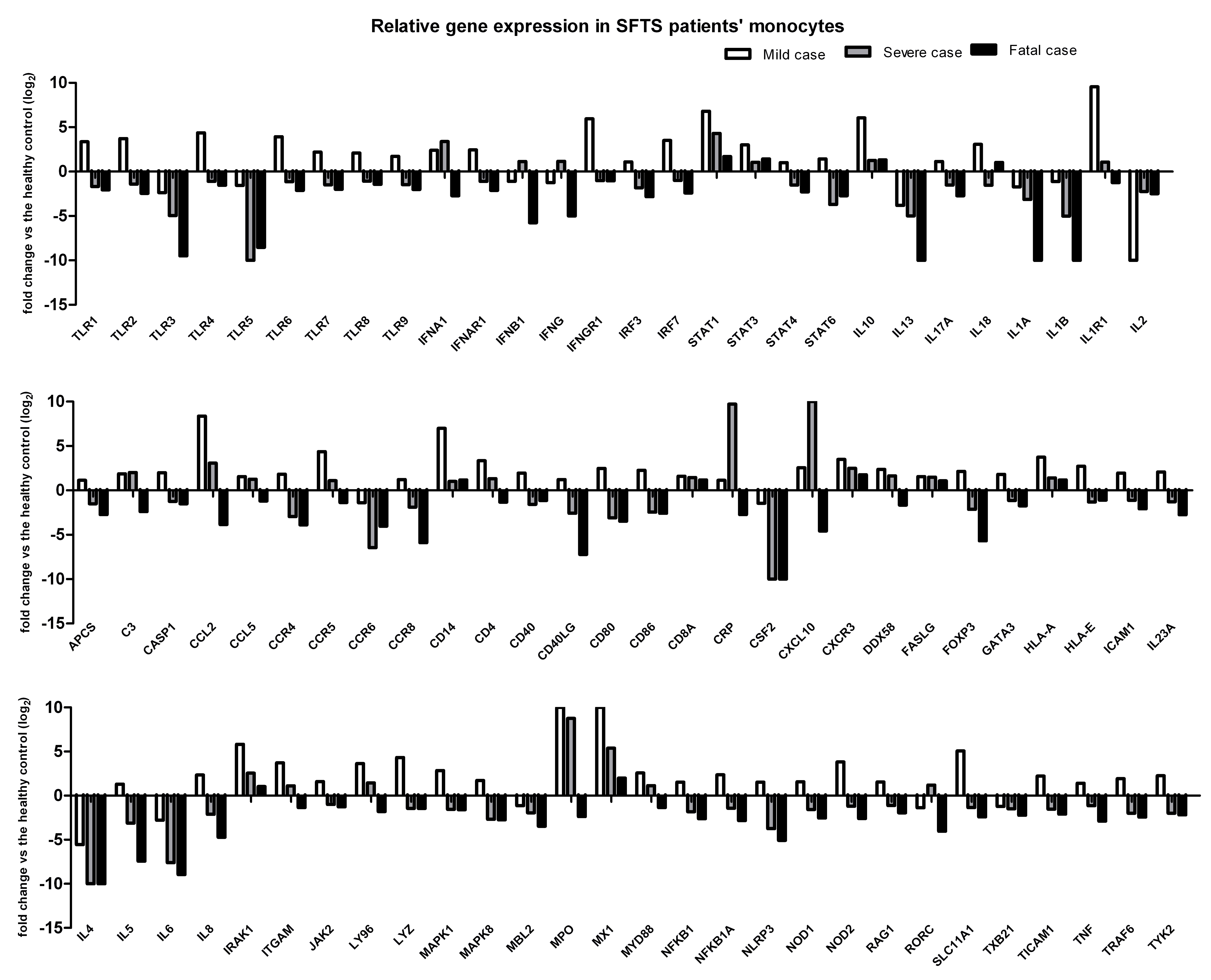


**Figure S2. Measurement of relative expression of 84 genes involved in innate and adaptive immune response in peripheral monocytes of SFTS patients during acute phase.**


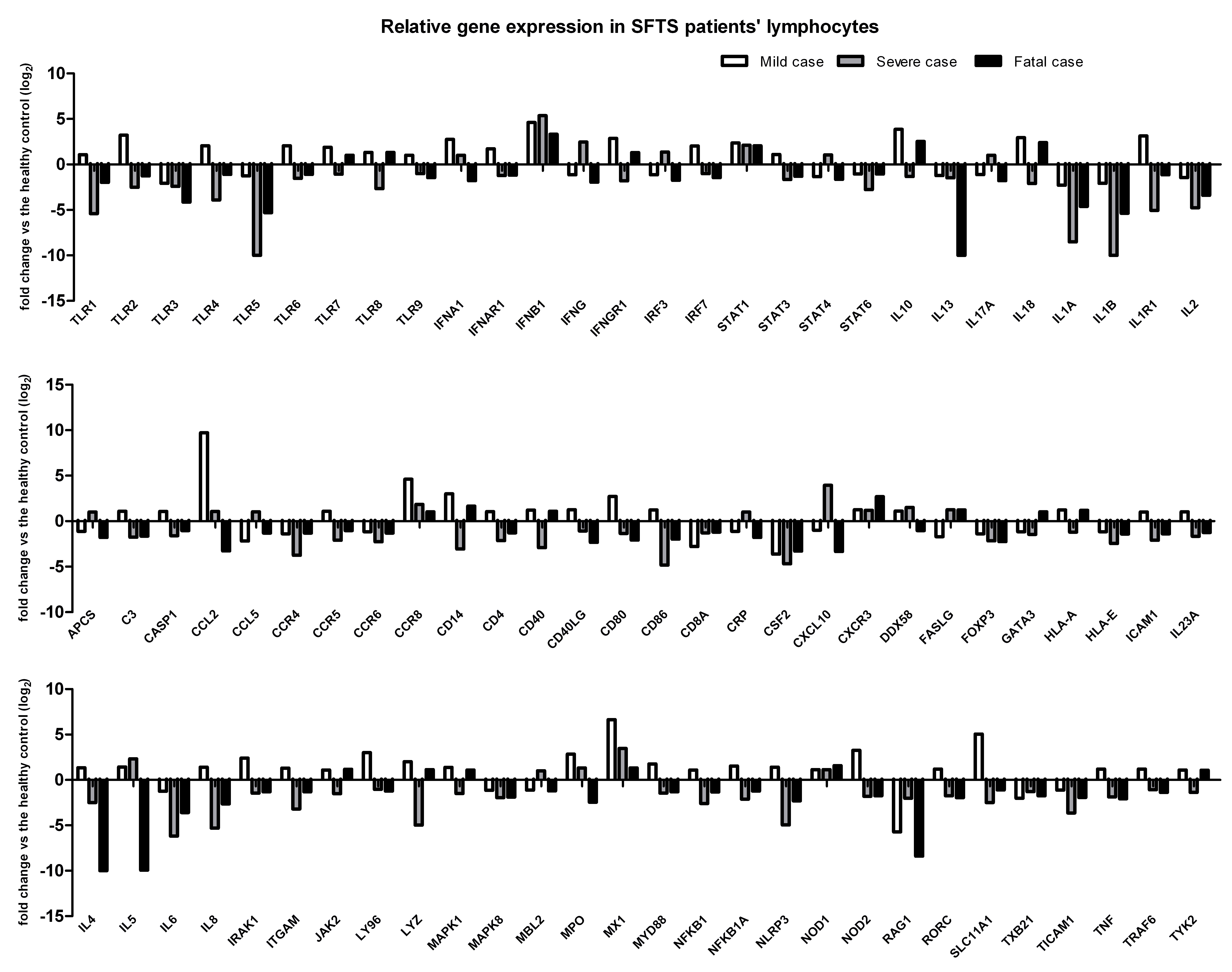


**Figure S3. Measurement of relative expression of 84 genes involved in innate and adaptive immune response in peripheral lymphocytes of SFTS patients during acute phase.**


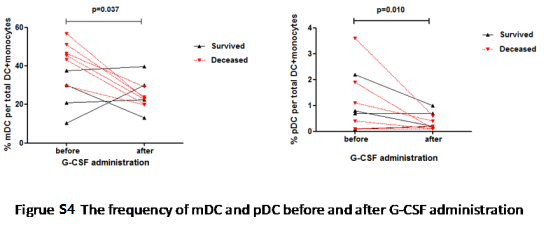


**Supplementary Tables**

Table S1. Group information and clinical complications of the SFTS patient cohort

| Group^†^ | n | Age range (median age) | Gender (M/F) | Complication^‡^ incidence | | | | | | | |
| --- | --- | --- | --- | --- | --- | --- | --- | --- | --- | --- | --- |
|  |  |  |  | Encephalitis | Hemorrhage | Respiratory failure | | Renal failure | | Hepatic failure | Pancreatitis |
| Mild | 44 | 26-77(56.2) | 21/23 | 0 | 0 | | 0 | | 0 | 0 | 0 |
| Severe | 18 | 43-73(59.8) | 10/8 | 9/18 | 6/18 | | 2/18 | | 1/18 | 0 | 0 |
| Fatal | 8 | 60-78(64.0) | 4/4 | 4/8 | 6/8 | | 3/8 | | 3/8 | 1/8 | 1/8 |
| Total | 70 | 26-78(58.1) | 35/35 | 13/70 | 12/70 | | 5/70 | | 4/70 | 1/70 | 1/70 |

^†^ Grouping criteria:

mild group: recovered without any complications;

severe group: recovered with one or more complications;

fatal group: finally dead.

^‡^ Complication diagnosis criteria: related guidelines published by Chinese Medical Society

Table S2. Comparison of clinical parameters of different groups of SFTS patients

| Clinical parameters | Normal range | Mild (n=44) | Severe (n=18) | Fatal (n=8) | *p*_1-2_ ^†^ | *p*_1-3_ ^†^ | *p*_2-3_ ^†^ |
| --- | --- | --- | --- | --- | --- | --- | --- |
| WBC^‡^ count（10^9^/L） | 4-10 | 1.84(0.6-7.8) | 1.79(0.8-3.4) | 1.42(0.5-3.3) | ns | ns | ns |
| Platelet count（10^9^/L） | 100-300 | 42.0(11.0-96.0) | 30.4(8.0-63.0) | 22.8(10.0-50.0) | 0.009 | 0.002 | ns |
| APTT^‡^（s） | 20-40 | 44.9(21.6-139.1) | 70.0(35.0-234.0) | 94.3(51.5-179.0) | 0.010 | 0.000 | ns |
| PT^‡^（s） | 10-15 | 12.2(10.0-15.2) | 15.7(10.5-41.9) | 17.2(13.1-31.0) | 0.004 | 0.003 | ns |
| TT^‡^（s） | 13-21 | 25.5(14.4-110.0) | 44.3(12.0-110.0) | >120(n=6 )^§^ | 0.002 | - | - |
| ALT^‡^（U/L） | 5-40 | 121.8(21.0-440.7) | 120.4(22.0-286.0) | 509.2(85.0-1668.2) | ns | 0.000 | 0.000 |
| LDH^‡^（U/L） | 109-245 | 1386.0(252.0-10750.0) | 1541.2(555.0-4560.0) | 8678.7(4670.0-18040.0) | ns | 0.000 | 0.000 |
| CK^‡^（U/L） | 30-170 | 730.8(26.0-7426.0) | 995.9(125.0-3545.0) | 2888.5(513.0-6693.0) | ns | 0.000 | 0.003 |
| Serum AMS^‡^（U/L） | 30-110 | 177.8(30.0-702.0) | 388.3(47.0-1440.0) | 323.8(135.0-690.0) | 0.003 | ns | ns |
| Serum Cr^‡^（μmol/L） | 39-142 | 60.4(39-142) | 64.8(38-109) | 177.8(64-413) | ns | 0.000 | 0.000 |

^†^ *p*1-2, *p*1-3, and *p*2-3 represent statistical significance of mild-severe, mild-fatal and severe-fatal pairs, respectively;

^‡^ Abbreviation of clinical parameters: WBC, peripheral white blood cells; APTT, activated partial thromboplastin time; PT, prothrombin time; TT, thrombin time; ALT, alanine aminotransferase; LDH, lactate dehydrogenase; CK creatine kinase; AMS amylase; Cr creatinine.

^§^ TT of 6 patients in the fatal group were beyond he detection limit.

Table S3. Correlation analysis of 2 variables between serum cytokine level and flow cytometry data of DCs and monocytes population and TLR3 expression in them.

|  | mDC | pDC | TLR3 in mDC | TLR3 in pDC | TLR3 in monoycte |
| --- | --- | --- | --- | --- | --- |
| IFN-α | -0.153^a^ (0.531^b^) | -0.160 (0.511) | 0.429 (0.066) | 0.446 (0.055) | 0.247 (0.306) |
| IFN-β | -0.328 (0.170) | 0.045 (0.854) | 0.513 (0.024)^*^ | 0.526 (0.020) ^*^ | 0.367 (0.121) |
| IFN-γ | 0.132 (0.587) | -0.281(0.243) | 0.332 (0.164) | -0.007(0.974) | 0.264 (0.274) |
| IL-28a | 0.025 (0.918) | 0.168 (0.490) | -0.235(0.331) | -0.191(0.431) | -0.218 (0.369) |
| IL-28b | 0.116 (0.635) | -0.258 (0.284) | -0.240 (0.321) | -0.268 (0.266) | -0.302 (0.207) |
| IL-29 | 0.086 (0.724) | -0.058 (0.813) | -0.139 (0.569) | 0.226 (0.350) | -0.018 (0.940) |
| IL-1β | -0.359 (0.130) | 0.090 (0.712) | -0.280 (0.509) | -0.037 (0.879) | -0.605 (0.006) ^*^ |
| IL-6 | 0.837(0.000) ^*^ | -0.053(0.826) | 0.587 (0.008) ^*^ | -0.274(0.256) | 0.523(0.021) ^*^ |

^a, b^ For each correlation analysis by Pearson test, ^a^ represents correlation coefficient (r value) and ^b^ P value of significance, and P<0.05 is considered as significant.

* represents strong significant correlation between the two indicated parameters which is designated by r>0.5 or <-0.5, and P<0.05.

Table S4. Multivariable linear regression analysis to evaluate the impact of multi-cytokines on the circulating DC populations and TLR3’s expression in them.

|  | mDC | pDC | TLR3 in mDC | TLR3 in pDC | TLR3 in monoycte |
| --- | --- | --- | --- | --- | --- |
| IFN-α | 0.004 ^a^ (0.401 ^b^) | -0.001(0.010) | -2.222(0.606) | -5.479(0.282) | -11.88(0.043) |
| IFN-β | -0.014(0.222) | 0.002(0.019) | 30.23(0.010) ^*^ | 23.00(0.064) | 43.23(0.004) |
| IFN-γ | -0.000(0.893) | -0.000(0.140) | -0.921(0.222) | -1.032(0.235) | -1.396(0.141) |
| IL-28a | 0.009(0.238) | 0.000(0.665) | 3.516(0.576) | -6.549(0.373) | 2.724(0.724) |
| IL-28b | -0.000(0.848) | -0.000(0.011) | -0.279(0.849) | -4.578(0.019) | -4.993(0.018) |
| IL-29 | 0.014(0.632) | 0.004(0.042) | 1.983(0.938) | 93.10(0.009) ^*^ | 84.82(0.020) ^*^ |
| IL-1β | 0.001(0.365) | 0.000(0.466) | -3.484(0.054) | -1.848(0.340) | -3.873(0.078) |
| IL-6 | 0.001(0.000) | 0.000(0.206) | 0.687(0.008) | -0.253(0.319) | 0.737(0.017) |

^a, b^ For multivariable linear regression analysis, ^a^ represents regression coefficient of serum cytokine level to circulating DC populations and TLR3’s expression in them and ^b^ P value of significance, and P<0.05 is considered as significant.

* represents the impact of the cytokine to the largest extent on the DC populations and TLR3’s expression in them.
